# Little Seïd

**Published:** 2007-10-22

**Authors:** David Ponka

Little Seïd is going home today, but he almost didn’t make it. He had seemed healthy at birth; his parents were thrilled, having lost their three other young children. But he had the misfortune to be born here, in the region where Chad and Sudan meet along a border interwoven by conflict and misery, and his family have been on the move for months. Seïd doesn’t have a home: he lives under a standard-issue, white and blue High Commission for Refugees tarpaulin that appeared from the sky one day.

The winds can be remorseless here. It makes the sand infiltrate everything, everywhere, even the decent straw hut that I share with the staff behind our little field hospital. When our youngest patients start crying at sunrise we wake up, notice the grit between our teeth and spit it out. But babies are rarely born with teeth, and they certainly can’t spit.

Seïd’s parents brought him to us a week ago because he wouldn’t breastfeed. A quick look was enough to tell me he was in trouble: he was breathing quickly, very quickly, and his chest caved and crushed him with every strained and ineffective breath. He also had a fever. This was bad news: he had bronchiolitis, a viral infection that normally takes hold of a baby’s lungs and doesn’t let go for three or four days. In the state little Seïd was in, I wasn’t sure he had three or four hours.

We devised a sort of spacing device from some used pill containers to give him some Ventolin, injected him with steroids and adrenalin and looked at each other and his mother, knowing this would likely not be enough. Back home we would intubate and ventilate him, or at least give him oxygen. We were overdue for a massive shipment from our Médecins Sans Frontières headquarters and knew we had asked for an oxygen concentrator. But night was falling, and with a pang of anxiety we realized that the shipment would not arrive today — whether because of bad roads or a hijacking, we didn’t know.

I placed Seïd in the position that he seemed least uncomfortable in and crawled off to bed, but neither of us slept that night. The heat had been stifling that day, and all the frogs and crickets that had sought refuge in our straw hut were croaking and creaking. Besides, I could hear little Seïd choking next door. All night I thought of going for a walk to get some fresh desert air, but I worried that I would step on a frog and crush it.

In the morning, Seïd was still alive, but barely. We gave him more medication, but he was starting to breathe irregularly, his fragile chest muscles too starved to keep going. I gave him some sugar water, hoping he was old enough to respond to placebo. Just then, miraculously, our truck pulled in. Our driver couldn’t understand, at first, why we were so glad to see him.

It was a large shipment; it took a frantic hour to find the right boxes and put everything together. By that time Seïd had stopped breathing. But he still had a heartbeat. We gave a couple of breaths and some more adrenalin and started our generator and the concentrator. He improved, but we were worried: our single generator needed a break every four hours, but babies need to breathe all the time. This baby would need another two or three days before he could breathe on his own.

That night, I had a nightmare. I dreamt that a child fell violently off a tree and broke his neck. I wanted to make sure he did not move, but someone came along and — out of mercy, it seemed — broke the child’s neck fully. I woke up in a sweat; little Seïd was still gasping for life.

All week I worried about that baby. It was an exceptionally busy week in this region where malaria is so prevalent that the word for any medicine translates as “quinine,” where home births and thus neonatal tetanus are not the exception but the rule, and where poverty and displacement make typhoid and other water- and food-borne illness all the more common. We even treated some gunshot wounds that I would rather not talk about. And yet, there were regular moments of solace, too: the bucket shower every morning, waving to healthy children who screamed “The white man! The white man!” during the midday truck ride to the outpatient clinic, the moment of peace before slumber when anything — even miracles — seemed possible.

During the second night there was a violent thunderstorm — but no rain. The morning felt strangely heavy and anticlimactic, but I convinced myself that little Seïd looked a little better.

The third night was as clear and crisp as a midnight dewdrop in the dry desert. I had another strange dream, one of the many I’d been having since my arrival here. This time I was back home, watching a man and his son enjoying their manicured back yard — the man floating in his swimming pool, the child washing his purebred dog with a breed-specific shampoo. I was holding Seïd in my arms, but the man and his son did not notice us.

And in the morning I ran into the hospital and saw that Seïd was crying as babies should and breastfeeding and I too started to cry. Crying is another thing I’ve been doing lot of here.

Now, I watch Seïd and his parents return into the desert and I wonder what will happen to them. They are no better off than they were before, but there is still a baby to feed, and shelter, and hope for. But Seïd’s parents do not seem worried. People here do not have much left, but they do still have something very powerful. *Inshallah*, they say, having faith in something that they face at least five times a day and beside which, they realize, they are small and powerless. God, at least, has not abandoned them.

I am not usually a religious man — and yet this week, in this seemingly empty desert, despite the pace and the violence and the hopelessness, or maybe because of it, little Seïd has taught me to pray.

